# Successful conservative management with uterine preservation in an adolescent with Couvelaire uterus and incomplete HELLP syndrome: a case report

**DOI:** 10.3389/fmed.2025.1666841

**Published:** 2025-12-05

**Authors:** Alice Gaibor-Pazmiño, Alex Oña, Ana Claudia Mendes Barbosa, Esteban Ortiz-Prado, Juan S. Izquierdo-Condoy

**Affiliations:** 1 Departamento de Ginecología y Obstetricia, Hospital Pablo Arturo Suárez, Quito, Ecuador; 2 One Health Research Group, Universidad de las Americas, Quito, Ecuador; 3 Faculdade de Medicina, Centro Universitario Várzea Grande, Cuiabá, Brazil

**Keywords:** Couvelaire uterus, HELLP syndrome, preeclampsia, placental abruption, adolescent pregnancy

## Abstract

**Introduction:**

Couvelaire uterus (uteroplacental apoplexy) is a rare, life-threatening obstetric emergency caused by severe placental abruption. It involves hemorrhagic infiltration of the myometrium and uterine serosa and is typically diagnosed intraoperatively. The condition is closely linked to hypertensive disorders of pregnancy, particularly HELLP syndrome. Although conservative management may preserve fertility, it is often complicated by massive postpartum hemorrhage requiring emergency hysterectomy.

**Case presentation:**

We report the case of a 19-year-old primigravid adolescent at 35.3 weeks of gestation who presented to a secondary-level hospital with sudden-onset abdominal pain and absent fetal movements. She had attended only four prenatal visits and had no prior comorbidities. On admission, she was hypertensive with hyperreflexia and marked proteinuria. Laboratory tests indicated severe preeclampsia with incomplete HELLP syndrome. Fetal demise was confirmed, and an emergency cesarean section was performed. Intraoperative findings revealed an 80% placental abruption, a 1,000 mL retroplacental hematoma, and diffuse uterine ecchymosis consistent with Couvelaire uterus. Uterine atony with an estimated blood loss of 2000 mL was successfully controlled using a modified B-Lynch compression suture and bilateral uterine artery ligation. Postoperatively, the patient developed hypovolemic shock and KDIGO stage II acute kidney injury requiring intensive care but achieved full recovery and was discharged on postoperative day 5.

**Conclusion:**

This case illustrates the diagnostic challenges of Couvelaire uterus in hypertensive pregnancies, particularly among adolescents with limited prenatal care, where placental abruption may occur silently and HELLP syndrome may present incompletely. The successful control of massive hemorrhage and preservation of the uterus using conservative techniques underscore the feasibility of fertility-sparing management even in resource-limited settings. Strengthening access to prenatal and emergency obstetric care remains essential to prevent adverse outcomes in vulnerable populations.

## 1 Introduction

Couvelaire uterus is a rare obstetric complication that arises from severe placental abruption of a normally inserted placenta. It is characterized by extravasation of blood into the myometrium, uterine serosa, and occasionally the peritoneal cavity (1–3). While most commonly reported in association with severe preeclampsia, HELLP syndrome (frequently accompanied by coagulopathy), and intrauterine fetal demise, its exact incidence is difficult to ascertain but is estimated to complicate <1% of abruptions and is not exclusively linked to older multigravidas; cases are well-documented across various age groups and parities, including young primigravids (2, 4–9). Intraoperatively, the uterus typically displays a violaceous discoloration with increased but ineffective tone, and may present with overt or concealed hemorrhage, as described in several case reports (2, 4, 5, 10). This condition reflects massive decidual bleeding and constitutes an obstetric emergency in which intraoperative recognition and timely management are essential to reduce high maternal morbidity (up to 43%) and perinatal mortality (14.1%) (6, 7, 11).

Hypertensive disorders of pregnancy—particularly severe preeclampsia and HELLP syndrome, including its incomplete variants—are key predisposing factors in the development of this condition. These entities share pathophysiological mechanisms such as widespread endothelial dysfunction, activation of the coagulation cascade, and microvascular injury, which collectively contribute to extensive placental detachment and hemorrhagic infiltration of the myometrium (1, 12). Incomplete or subclinical HELLP syndrome often delays diagnosis, exacerbating risks of complications like acute renal failure, disseminated intravascular coagulation, and cerebral hemorrhage. The evidence on this association is limited to isolated case reports, underscoring its low prevalence but significant clinical relevance (2, 4, 13, 14). Conservative strategies such as oxytocin infusion, uterine compression sutures, and uterine packing can enable uterine preservation in selected cases (3, 4, 12). However, severe cases may necessitate hysterectomy, particularly when associated with unresponsive uterine atony or major intra-abdominal bleeding (4).

This report presents an uncommon clinical case of Couvelaire uterus in a 35.1-week primigravid adolescent, associated with severe preeclampsia and incomplete HELLP syndrome. It highlights diagnostic challenges, conservative surgical decision-making, and the importance of early recognition, particularly in settings with limited access to prenatal care.

## 2 Case presentation

We report the case of a 19-year-old mestiza adolescent, primigravida, with no relevant past medical history, who presented to the obstetric emergency department at 35.3 weeks of gestation based on her last menstrual period (LMP). She reported lower abdominal cramping pain radiating to the lumbar region, with a duration of approximately four hours, accompanied by absent fetal movements. She denied loss of amniotic fluid, vaginal bleeding, headache, blurred vision, or fever.

She had attended four prenatal visits during the pregnancy, and two previous obstetric ultrasounds had revealed no abnormalities.

At admission, her blood pressure was elevated (136/101 mmHg), with brisk deep tendon reflexes and significant proteinuria (2088 mg/dL), with a protein-to-creatinine ratio of 18.48. Laboratory testing showed elevated lactate dehydrogenase (LDH 635 IU/L), while hepatic enzymes (AST 39.2 IU/L; ALT 15.2 IU/L) and platelet count (153,000/μL) were within normal limits. These findings, along with hypertension and heavy proteinuria, were consistent with severe preeclampsia and incomplete HELLP syndrome (Table 1).

**Table 1:** Structured clinical timeline.

Timepoint	Date/time	Event	Vitals	Lab results	Interventions	EBL (mL) and blood products	Notes
T0	30/08/2023 ~ 18:30	Symptom onset: colicky abdominal pain, no fetal movement	–	–	–	–	First symptoms noted by patient
T1	30/08/2023 23:35	Hospital admission	BP 136/101, HR 80, RR 20, T 36.2 °C, SpO₂ 94%	LDH 635 IU/L (ref 140–280 IU/L), Plt 153 × 10^3^/μL (ref 150–400 × 10^3^/μL), AST 39.2 IU/L (ref < 35 IU/L), ALT 15.2 IU/L (ref < 35 IU/L), Cr 1.29 mg/dL (ref 0.5–1.0 mg/dL), Prot/Cr 18.4 (ref < 0.3), Urine protein 2088 mg/dL (severely elevated), Alkaline phosphatase 202 IU/L (ref 44–147 IU/L).	MgSO₄ 4 g bolus + 1 g/h (in 880 mL SSN at 40 mL/h), Cefazolin 1 g IV q6h x 3 doses, Omeprazole 40 mg IV daily	–	Severe preeclampsia + fetal demise; initial labs and prophylaxis
T2	31/08/2023 00:30	Surgical decision	BP 157/105, HR 76, RR 18, T 36.3 °C, SpO₂ 92%	Hb 11.5 g/dL (ref 12–16 g/dL), Hct 35.8% (ref 36–46%), Plt 153 × 10^3^/μL (ref 150–400 × 10^3^/μL), Leu 20.82 × 10^3^/μL (ref 4–11 × 10^3^/μL), Neut 17.34 × 10^3^/μL (ref 1.5–8 × 10^3^/μL), Neut% 83.3 (ref 40–75%).	Pre-op prep; anesthesia clearance	–	Decision for emergency cesarean
T3	31/08/2023 01:20–02:30	Cesarean section + hemostatic sutures	BP 117/69, HR 64, RR 10, T 36.8 °C, SpO₂ 90%	–	Modified B-Lynch, bilateral uterine artery ligation, 1 g TXA IV, 1 PRBC unit	2000 ml 1 PRBC intraop	Couvelaire uterus, 80% abruption, dead fetus (2,160 g), TXA administered
T4	31/08/2023 03:45	ICU admission	BP 110/65, RR 13, T 36.1 °C, SpO₂ 93%	Hb 9.5 g/dL (ref 12–16 g/dL) Hct 27.7% (ref 36–46%) Plt 82 × 10^3^/μL (ref 150–400 × 10^3^/μL) LDH 844 IU/L (ref 140–280 IU/L) AST 57 IU/L (ref < 35 IU/L) Cr 1.29 mg/dL (ref 0.5–1.0 mg/dL)	MgSO₄ dose halved and later suspended due to toxicity (arreflexia, oliguria <0.1 mL/kg/h), Oxytocin 20 IU in RL 1000 mL (40 mL/h)	2 PRBC (08:45), 1 FFP (10:15), 5 Platelets, 7 Cryoprecipitate	Conservative hemodynamic and renal management in ICU
T5	01/09/2023 a.m.	Hematologic recovery phase	BP 135/80, HR 82, RR 18, T 36.5 °C, SpO₂ 90%	Peripheral smear: anisocytosis (+); Seg 94%; Plt 122 × 10^3^/μL (ref 150–400 × 10^3^/μL); Hb 11.2 g/dL (ref 12–16 g/dL); Hct 34.3% (ref 36–46%); Leu 21.99 × 10^3^/μL (ref 4–11 × 10^3^/μL); Neut 9.90 × 10^3^/μL (ref 1.5–8 × 10^3^/μL); Uric acid 8.28 mg/dL (ref 2.6–6.0 mg/dL).	Meropenem initiated for suspected infection	–	Antimicrobial escalation and follow-up labs
T6	02/09/2023	Drop in hematologic parameters	BP 135/88, HR 93, RR 20, T 36.5 °C, SpO₂ 93%	Hb 7.3 g/dL (ref 12–16 g/dL), Hct 22.4% (ref 36–46%), LDH 328 IU/L (ref 140–280 IU/L).	–	–	Ongoing hemolysis, monitored conservatively
T7	03/09/2023	Lactation suppression	BP 117/84, HR 104, RR 20, T 37 °C, SpO₂ 96%	–	Cabergoline 0.25 mg PO q12h x 1 day	–	Suppression of milk production
T8	04/09/2023	Continued anemia	BP 119/86, HR 84, RR 18, T 36.4 °C, SpO₂ 92%	Hb 7.9 g/dL (ref 12–16 g/dL), Hct 24.9% (ref 36–46%).	–	–	Slow hematologic recovery
T9	05/09/2023	Ongoing recovery, anemia moderate	BP 111/63, HR 66, RR 20, T –, SpO₂ 90%	Hb 8.6 g/dL (ref 12–16 g/dL), Hct 26.7% (ref 36–46%), Plt 165 × 10^3^/μL (ref 150–400 × 10^3^/μL).	Ampicillin/sulbactam PO, iron saccharate IV, NSAIDs, wound care, nifedipine PRN	–	Hemodynamically stable, no signs of infection
T10	06/09/2023	Hospital discharge	BP 93/72, HR 69, RR 20, T 36.5 °C, SpO₂ 92%	Same as T9	Full oral medications + follow-up	–	Discharged stable with contraception and outpatient plan

BP, blood pressure; HR, heart rate; RR, respiratory rate; T, temperature; SpO₂, peripheral oxygen saturation; LDH, lactate dehydrogenase; Plt, platelets; AST, aspartate aminotransferase; ALT, alanine aminotransferase; Cr, creatinine; Prot/Cr, urine protein-to-creatinine ratio; MgSO₄, magnesium sulfate; SSN, normal saline solution; Hb, hemoglobin; Hct, hematocrit; Leu, leukocytes; Neut, neutrophils; TXA, tranexamic acid; PRBC, packed red blood cells; FFP, fresh frozen plasma; EBL, estimated blood loss; RL, Ringer’s lactate; ICU, intensive care unit; PO, per os (oral route); IV, intravenous; PRN, as needed; NSAIDs, non-steroidal anti-inflammatory drugs.

Obstetric evaluation revealed a singleton fetus in cephalic presentation, with uterine height appropriate for gestational age. Fetal monitoring showed no cardiac activity or uterine contractions. An emergency ultrasound confirmed intrauterine fetal demise. On vaginal examination, the cervix was soft, posterior, 1 cm dilated, and 30% effaced.

Given the context of severe preeclampsia, laboratory findings consistent with incomplete HELLP syndrome, and fetal demise, an emergency cesarean section was indicated. Magnesium sulfate was administered for maternal neuroprotection, and preparations were made to terminate the pregnancy.

**Figure 1:**
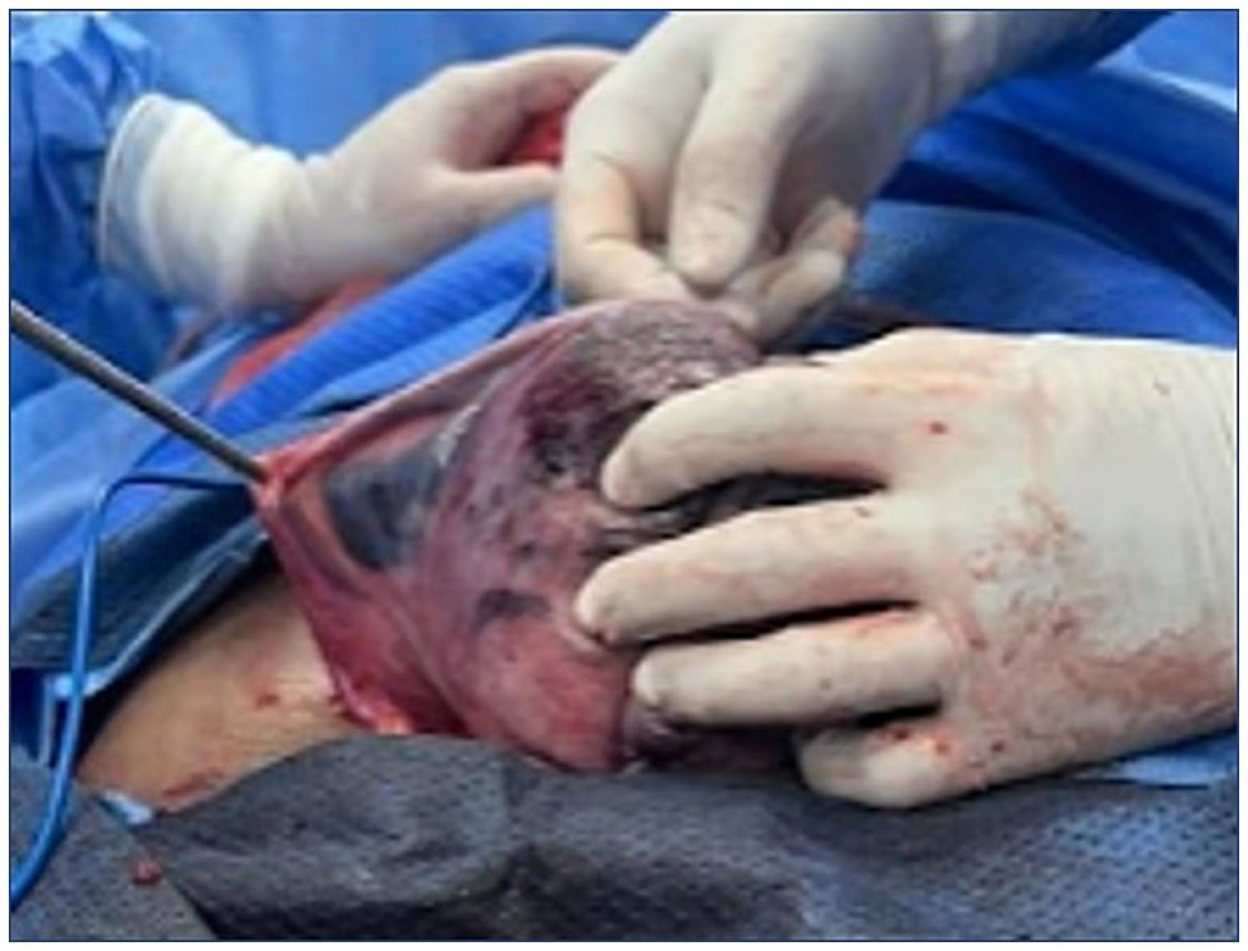
Hemorrhagic infiltration of the myometrium in Couvelaire uterus. The image displays the uterus during cesarean section with marked serosal ecchymosis and violaceous discoloration caused by blood dissecting through the myometrium and serosa. This macroscopic appearance confirms Couvelaire uterus, characterized by hemorrhagic extension beyond the placental bed into the uterine wall and potentially the peritoneal surface. The photograph was obtained intraoperatively with written informed consent from the patient’s legal guardian. The image has been fully de-identified.

During spinal anesthesia-assisted cesarean delivery, the uterus appeared gravid with extensive ecchymotic infiltration involving approximately 40% of the anterior wall and 20% of the right broad ligament, consistent with Couvelaire uterus (Figure 1). The amniotic fluid was dark and wine-colored. An estimated 80% placental abruption and a retroplacental hematoma measuring approximately 1,000 mL were identified. A stillborn female fetus weighing 2,160 g was delivered. Histopathological analysis of the placenta confirmed acute suppurative chorioamnionitis, decidual and intraplacental hemorrhage, and ischemic villous changes. Microscopy revealed necrosis, fibrinoid deposition, and extensive intervillous hemorrhage, along with inflammation of the fetal membranes. The villous maturation was consistent with 34–35 weeks of gestation. These pathological findings corroborate the clinical diagnosis of placental abruption and intrauterine fetal demise, further explaining the maternal decompensation observed perioperatively.

**Figure 2:**
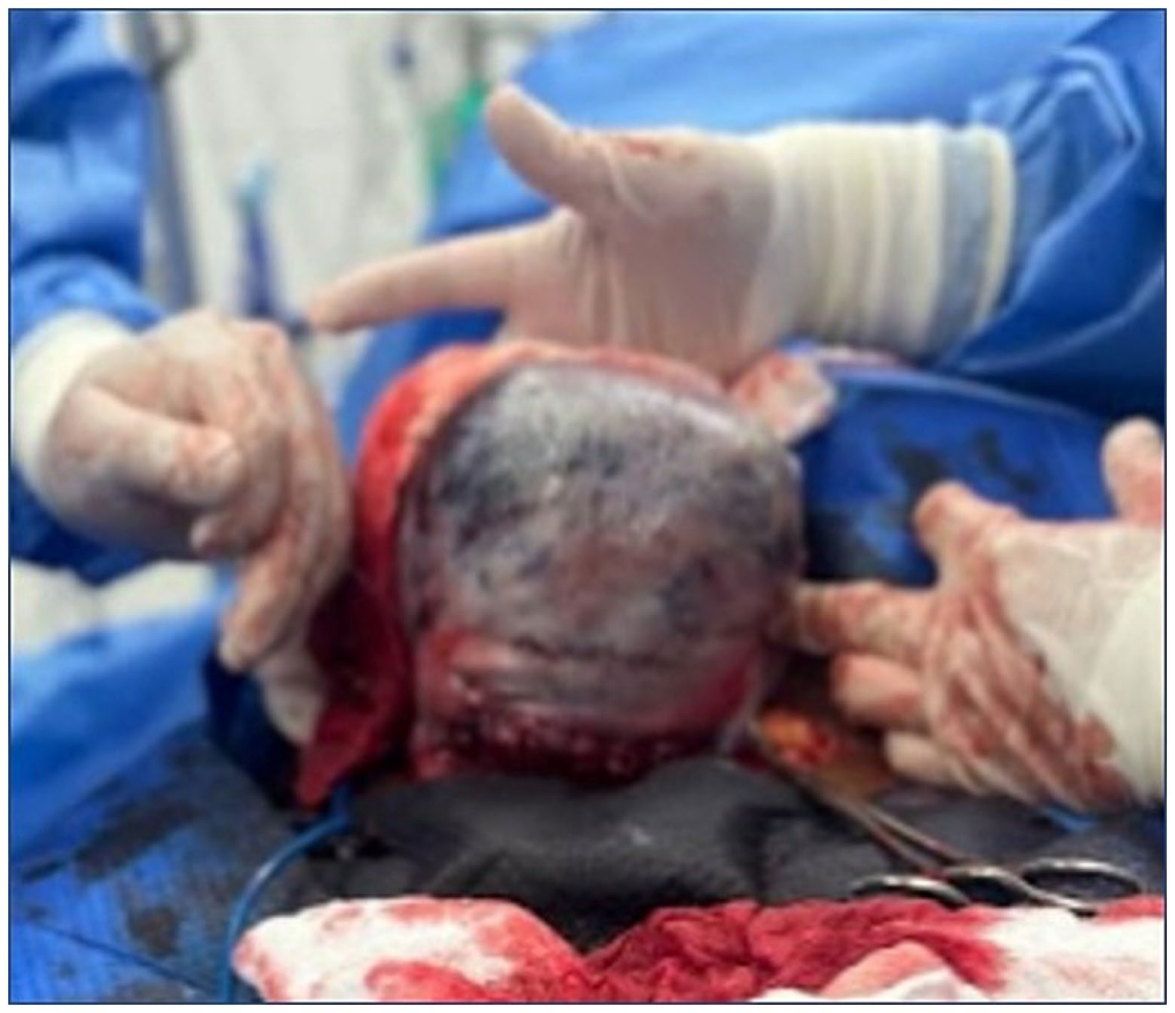
Macroscopic appearance of Couvelaire uterus. This intraoperative view shows the exteriorized uterus with characteristic violaceous marbling and patchy subserosal hemorrhage, reflecting diffuse myometrial blood infiltration secondary to placental abruption. The mottled discoloration pattern illustrates the classic gross manifestation of uteroplacental apoplexy, highlighting the extent of hemorrhagic spread within the uterine wall. The photograph was obtained intraoperatively with written informed consent from the patient’s legal guardian. The image has been fully de-identified.

**Figure 3:**
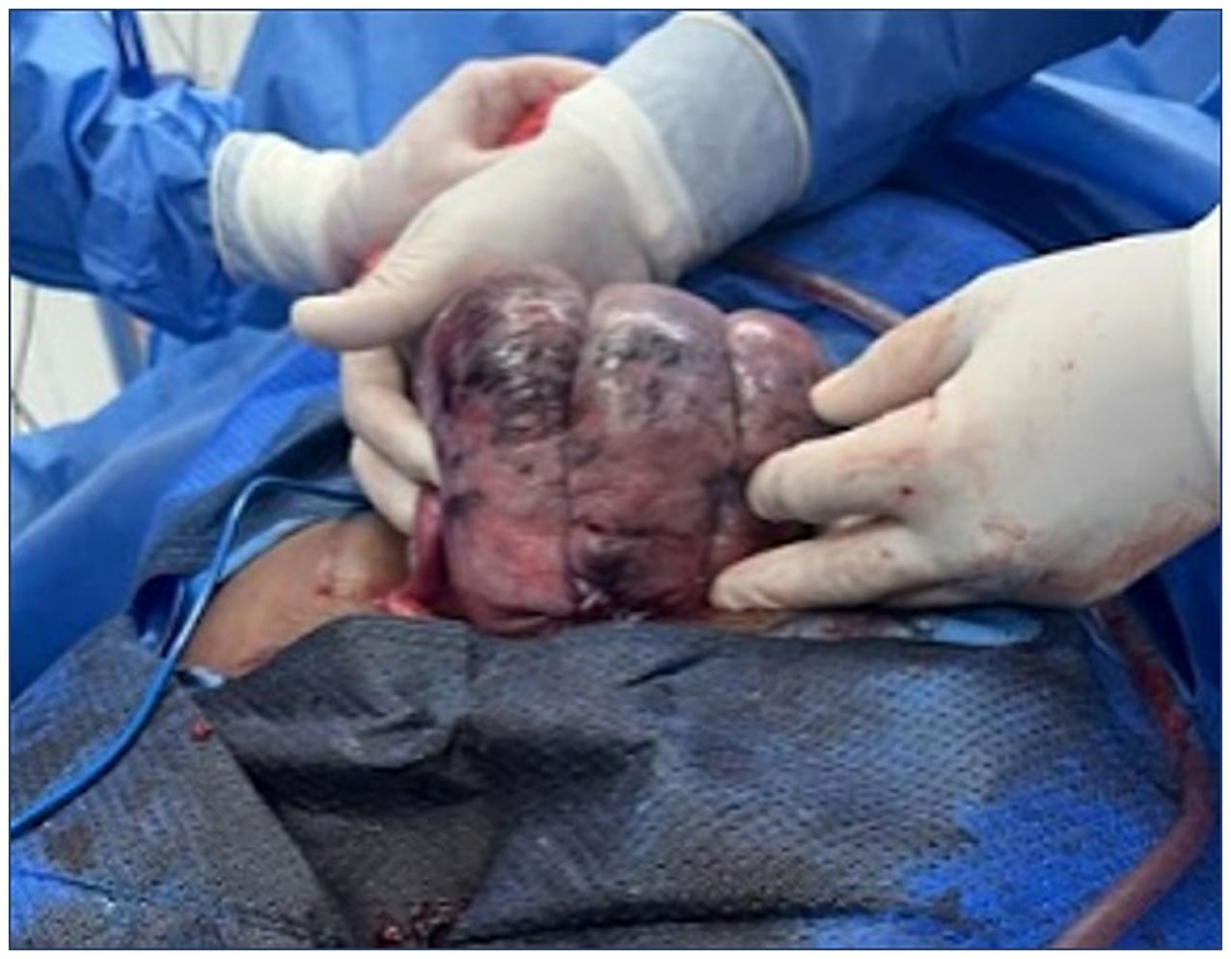
Application of B-Lynch compression suture. This intraoperative image highlights the placement of a B-Lynch compression suture as a uterus-preserving strategy for the control of postpartum hemorrhage secondary to uterine atony. The vertical compression technique demonstrates mechanical stabilization of the uterine body aiming to restore tone and achieve effective hemostasis. The photograph was obtained intraoperatively with written informed consent from the patient’s legal guardian. The image has been fully de-identified.

The patient developed uterine atony with an estimated blood loss of 2000 mL, which was successfully controlled using a modified B-Lynch compression suture, thereby avoiding hysterectomy (Figures 2, 3). In the immediate postoperative period, the patient developed hypovolemic shock secondary to severe obstetric hemorrhage and was transferred to the intensive care unit (ICU). She exhibited acute kidney injury (KDIGO stage II) characterized by oliguria (<0.5 mL/kg/h) and mild metabolic acidosis. Estimated blood loss (EBL) was 2000 mL, assessed by visual estimation and suction canister volume. Hemostatic management included intraoperative administration of 1 g tranexamic acid (TXA) IV and immediate transfusion of 1 unit of packed red blood cells (PRBC). Subsequently, in the ICU, she received 2 more PRBC units (08:45), 1 unit of fresh frozen plasma (10:15–10:45), 5 platelet concentrates in the afternoon, and 7 cryoprecipitates in the evening. ROTEM/TEG monitoring was not available; therefore, transfusion thresholds followed the institutional massive transfusion protocol based on conventional laboratory parameters (hemoglobin <8 g/dL, platelets <100 × 10^3^/μL, INR > 1.2, and fibrinogen <200 mg/dL). Coagulopathy was anticipated based on persistent bleeding, declining hematologic parameters, and HELLP-related thrombocytopenia. Oxytocin 20 IU in 1000 mL RL at 40 mL/h was administered, followed by a modified B-Lynch compression suture and bilateral uterine artery ligation to control uterine atony and avoid hysterectomy. Broad-spectrum antibiotics were started with IV meropenem and later de-escalated to ampicillin-sulbactam. Rescue diuresis was achieved with IV furosemide, and glycemic control was maintained with crystalline insulin as per ICU protocol (Table 2).

**Table 2:** Sequential record of transfusion therapy and hemostatic parameters.

Component	Units	Pre-value	Post-value	Indication
PRBC	1 unit	Hb 11.5 → 9.5	–	Acute surgical bleeding (EBL 2000 mL)
PRBC	2 units	Hb 9.5	Hb 11.2 (01/09)	Anemia post-hemorrhage
FFP	1 unit	INR 1.13 Fibrinogen 532.6 mg/dL (ref 200–400 mg/dL).	INR 0.98 Fibrinogen ↓ to ~150 mg/dL due to ongoing hemorrhage	Coagulopathy prevention
Platelets	5 units	Plt 82 K	Plt 122 K (01/09)	HELLP-related thrombocytopenia
Cryoprecipitate	7 units	Fibrinogen 150 mg/dL (ref 200–400 mg/dL).	Fibrinogen 290 mg/dL (ref 200–400 mg/dL)	Hypofibrinogenemia suspected due to massive PPH
TXA	1 g	–	–	Antifibrinolytic, protocol for massive hemorrhage

PRBC, packed red blood cells; FFP, fresh frozen plasma; Hb, hemoglobin; Hct, hematocrit; Plt, platelets; INR, international normalized ratio; PPH, postpartum hemorrhage; TXA, tranexamic acid; EBL, estimated blood loss; HELLP, hemolysis, elevated liver enzymes, and low platelet count syndrome.

The clinical course was favorable, with progressive improvement in both clinical status and laboratory parameters. The patient was transferred to the general ward and discharged 5 days later, hemodynamically stable, with scheduled outpatient follow-up in gynecology and psychology.

The patient and her family were counseled regarding uterine preservation, fertility implications, and postpartum contraception, and written informed consent was obtained. During follow-up at 1-month, histopathologic results confirmed the diagnosis, and at 3 months she demonstrated complete clinical recovery with spontaneous return of normal menstruation. She was subsequently referred to primary care services for family planning counseling and long-term reproductive follow-up.

## 3 Discussion

This case demonstrates an atypical presentation of Couvelaire uterus in a primigravid adolescent with incomplete HELLP syndrome and extensive (DPPNI) placental abruption (80%). The intraoperative diagnosis—despite absent vaginal bleeding—highlights the critical need for anticipatory vigilance in hypertensive pregnancies exhibiting fetal compromise, particularly given that 10–20% of abruptions present covertly (1, 13).

Couvelaire uterus—characterized by hemorrhagic infiltration of the myometrium and uterine serosa—is typically diagnosed during surgical exploration and is strongly associated with severe placental abruption (1, 5). In this case, the patient presented with an extensive but clinically atypical manifestation of the condition, occurring in conjunction with incomplete HELLP syndrome (9). This combination is rarely reported, with only isolated case reports available, likely due to their shared but diagnostically elusive pathophysiology: microvascular endothelial damage that manifests as subclinical coagulopathy and silent myometrial extravasation (13, 15). The coexistence of these two entities significantly worsens the maternal-fetal outcomes by generating synergistic pathophysiological disruptions. These include heightened risks of multiorgan dysfunction, consumptive coagulopathy, and acute kidney injury—as seen in this patient, who developed KDIGO stage II AKI and thrombocytopenia (109,000/μL) (1, 2).

Despite an estimated blood loss of 2000 mL, uterine preservation was successfully achieved using a modified B-Lynch compression suture, thereby avoiding emergent peripartum hysterectomy, as previously described by Uwagbai et al. (14). Surgical decision-making in such contexts must be individualized, taking into account hemodynamic stability, the effectiveness of conservative measures, and the patient’s reproductive wishes. In this case, the uterus was already exteriorized and incised during cesarean delivery, facilitating the direct placement of the compression suture. Given the patient’s young age (19 years), stable coagulation profile, and desire for future fertility, the surgical team opted for uterine conservation. International guidelines (FIGO, ACOG) recommend uterine compression sutures in hemodynamically stable patients with controllable hemorrhage and favorable uterine tone response (16, 17). Hysterectomy is generally reserved for cases unresponsive to conservative measures or with rapid clinical deterioration. The patient responded promptly to the modified B-Lynch technique, with restoration of uterine tone and no further bleeding, supporting the decision to avoid hysterectomy in this scenario (18).

Historically, Couvelaire uterus was considered a surgical emergency warranting immediate hysterectomy due to its alarming appearance. However, recent data highlights that uterine preservation is both feasible and safe in hemodynamically stable patients. A retrospective study from Japan reported that none of the 12 patients with Couvelaire uterus required hysterectomy, although 58.3% received blood transfusions and 25% developed DIC, underscoring the value of early conservative intervention in selected cases (19). Comparatively, published data highlight the severe maternal and neonatal outcomes associated with abruptio placentae and Couvelaire uterus (20). One case series noted that about 5.8% of placental abruptions were complicated by coagulopathy and 16.8% by Couvelaire uterus. Patients with Couvelaire changes tend to have much higher blood loss and transfusion requirements than those without. For instance, Sunanda et al. reported that 67% of Couvelaire cases developed postpartum hemorrhage (versus ~29% in non-Couvelaire abruptions) and required an average of about 5–6 units of blood transfusion, significantly more than the ~2 units in controls. DIC occurred in 22% of Couvelaire cases (versus 14% without) and the maternal mortality in the Couvelaire group reached ~5.6% in that series (21). Taken together, these data reinforce that a Couvelaire uterus signals a high-risk situation often necessitating massive transfusion and critical care support.

Inadequate prenatal care evidenced by only four antenatal visits—was a critical factor delaying recognition of evolving pathology. This suboptimal follow-up obscured the progression of prodromal signs such as hypertension and proteinuria, postponing the diagnosis of both HELLP syndrome and occult placental abruption. Although the patient did not fulfill the full Mississippi criteria for classic HELLP syndrome—defined by hemolysis, elevated liver enzymes, and thrombocytopenia—her laboratory findings supported a diagnosis of incomplete HELLP. Hemolysis was evidenced by a marked LDH elevation (635 U/L), a drop in hemoglobin from 11.5 to 7.3 g/dL, and anisocytosis on peripheral blood smear. Liver enzymes were only mildly elevated (AST 39.2 U/L, ALT 15.2 U/L), and platelet count, initially normal (153,000/μL), declined to a nadir of 82,000/μL. This constellation meets criteria for the partial variant of HELLP syndrome, which—despite lacking one or more components of the classic triad—remains clinically significant and requires aggressive management due to its associated maternal risks (22, 23).

Alternative diagnoses were carefully considered. Acute fatty liver of pregnancy (AFLP) was deemed unlikely due to the absence of hypoglycemia, coagulopathy, and hepatic dysfunction. Thrombotic thrombocytopenic purpura (TTP) and atypical hemolytic uremic syndrome (aHUS) were ruled out given the lack of neurologic symptoms, normal creatinine clearance over time, and absence of schistocytes on smear (24–26). The diagnosis of incomplete HELLP was therefore established based on evolving hematologic trends, liver function, and clinical context. As a result, critical windows for early intervention—including enhanced blood pressure monitoring, corticosteroid administration, or timely delivery—were missed, increasing maternal and fetal risk (14, 20). Current guidelines underscore the importance of structured prenatal care: the American College of Obstetricians and Gynecologists (ACOG) recommends at least 12 visits for low-risk pregnancies, with biweekly assessments after 20 weeks to facilitate the early detection of preeclampsia and other complications (27).

Even with timely recognition and aggressive management, Couvelaire uterus remains associated with significant maternal morbidity. Potential complications include hypovolemic shock, disseminated intravascular coagulation (DIC), massive transfusion requirements, and postoperative uterine dysfunction—particularly in patients with hypertensive disorders or delayed presentations (7, 12, 14). Hemorrhagic infiltration of the myometrium impairs contractility, increasing the risk of refractory uterine atony and exacerbating hemorrhage (4).

Hysterectomy becomes a life-saving measure only when uterine atony remains unresponsive to maximal interventions and hemodynamic instability threatens multiorgan failure. Indicators for such intervention include transfusion of more than six units of blood products, serum lactate >4 mmol/L, or vasopressor dependence. Delaying hysterectomy beyond this threshold increases maternal mortality eightfold, while premature intervention may unnecessarily compromise future fertility (9).

In Latin America, many public and low-resource hospitals simply do not have access to ROTEM/TEG and must rely on conventional transfusion protocols. Consistent with this, a recent Argentine survey found that the vast majority of 55 specialized ICUs had no ROTEM/TEG device on site (28). Consequently, massive transfusion protocols (MTPs) in the region rely on fixed-ratio resuscitation (e.g., 1:1:1 PRBC:FFP:Platelets) and standard labs (INR, fibrinogen, Plt count) as outlined in regional guidelines (28). This patient’s transfusion decisions followed such protocols, guided by hemoglobin decline, persistent bleeding, and rising LDH.

From the fetal perspective, abrupt cessation of placental exchange often results in acute hypoxia and intrauterine death—as occurred in this case—despite prompt surgical intervention (2, 4, 20).

This case highlights the importance of early diagnosis, intensive monitoring, and a multidisciplinary approach in managing high-risk pregnancies—particularly among adolescents with limited access to prenatal care. It was prepared and reported in accordance with the CARE guidelines for case reports, ensuring transparency and reproducibility (29). Moreover, it contributes to the existing literature by demonstrating that even under critical clinical conditions, uterine preservation is feasible using conservative surgical strategies, thereby safeguarding maternal safety and future fertility (4, 5, 14).

## 4 Conclusion

Couvelaire uterus represents a rare but life-threatening obstetric emergency that typically becomes evident only during surgery. When it occurs in association with incomplete HELLP syndrome, as in this adolescent primigravid patient, the risk of severe maternal and fetal complications markedly increases. This case emphasizes the need for a high index of clinical suspicion for concealed placental abruption in hypertensive pregnancies—even in the absence of vaginal bleeding—and highlights how delayed or limited prenatal care can obscure early warning signs. Prompt multidisciplinary management, guided by standardized transfusion protocols and conservative surgical techniques such as the modified B-Lynch suture, allowed successful hemorrhage control and uterine preservation. Beyond its clinical relevance, this case provides evidence that fertility-preserving interventions are feasible and effective in well-selected patients, even under resource-limited conditions. Strengthening access to prenatal care and emergency obstetric services remains essential to prevent similar outcomes in vulnerable populations.

## Data Availability

The original contributions presented in the study are included in the article/supplementary material, further inquiries can be directed to the corresponding author.

## References

[1] Oyelese Y Ananth CV. Placental abruption. Obstet Gynecol. (2006) 108:1005–16. doi: 10.1097/01.AOG.0000239439.04364.9a, PMID: 17012465

[2] Hubbard JL Hosmer SB. Couvelaire uterus. J Am Osteopath Assoc. (1997) 97:536–7. doi: 10.7556/jaoa.1997.97.9.536 9313351

[3] Tikkanen M. Placental abruption: epidemiology, risk factors and consequences. Acta Obstet Gynecol Scand. (2011) 90:140–9. doi: 10.1111/j.1600-0412.2010.01030.x, PMID: 21241259

[4] Martínez LLT Gloria JPM Altamirano RDF Cruz GR Avalos OAL. A comprehensive review of Couvelaire uterus: diagnosis, and management. Int J Med Sci Clin Res Stud. (2024) 4:721–724. doi: 10.47191/ijmscrs/v4-i04-20

[5] Rathi M Rathi SK Purohit M Pathak A. Couvelaire uterus. BMJ Case Rep. (2014) 2014:bcr2014204211. doi: 10.1136/bcr-2014-204211, PMID: 24686812 PMC3975566

[6] Chen D Gao X Yang T Xin X Wang G Wang H . Independent risk factors for placental abruption: a systematic review and meta-analysis. BMC Pregnancy Childbirth. (2025) 25:351. doi: 10.1186/s12884-025-07482-7, PMID: 40140972 PMC11938633

[7] Mayo Clinic. Placental abruption - Symptoms & causes. (2022). Available online at: https://www.mayoclinic.org/diseases-conditions/placental-abruption/symptoms-causes/syc-20376458 (accessed June 23, 2025).

[8] Senkaya AR Umut F Ileri A Karaca SY Aytac H Oztekin DC. Maternal and neonatal outcomes of couvelaire uterus. Medicine (Baltimore). (2021) 10:775–8. doi: 10.5455/medscience.2021.02.059, PMID: 36448968

[9] Hiiragi K Obata S Miyagi E Aoki S. Clinical implications of a Couvelaire uterus with placental abruption: a retrospective study. Int J Gynaecol Obstet. (2025) 168:177–83. doi: 10.1002/ijgo.15821, PMID: 39056529

[10] Pritchard JA. Genesis of severe placental abruption. Am J Obstet Gynecol. (1970) 108:22–7. doi: 10.1016/0002-9378(70)90199-7, PMID: 5454580

[11] Miranda ML Vallejo-Vaz AJ Cerrillo L Marenco ML Villar J Stiefel P. The HELLP syndrome (hemolysis, elevated liver enzymes and low platelets): clinical characteristics and maternal–fetal outcome in 172 patients. Pregnancy Hypertens. (2011) 1:164–9. doi: 10.1016/j.preghy.2011.01.004, PMID: 26104498

[12] Kapesi V Moshi B Kyejo W Jusabani A Mgonja M Kaguta M. Couvelaire uterus in a previable pregnancy: Complication in abruptio placenta, case series from Tanzanian tertiary hospital. Int J Surg Case Rep. (2023) 102:107862. doi: 10.1016/j.ijscr.2022.107862, PMID: 36621218 PMC9850026

[13] Ananth CV Lavery JA Vintzileos AM Skupski DW Varner M Saade G . Severe placental abruption: clinical definition and associations with maternal complications. Am J Obstet Gynecol. (2016) 214:272.e1–9. doi: 10.1016/j.ajog.2015.09.069, PMID: 26393335

[14] Uwagbai ON Wittich AC. A 30-Year-Old Female Found to Have a Couvelaire Uterus With Placenta Accreta During Planned Cesarean Delivery. Mil Med. (2017) 182:e1877–9. doi: 10.7205/MILMED-D-16-00146, PMID: 28290978

[15] McNamara H Mallaiah S Barclay P Chevannes C Bhalla A. Coagulopathy and placental abruption: changing management with ROTEM-guided fibrinogen concentrate therapy. Int J Obstet Anesth. (2015) 24:174–9. doi: 10.1016/j.ijoa.2014.12.005, PMID: 25659517

[16] WHO Recommendations for the Prevention and Treatment of Postpartum Haemorrhage. Geneva: World Health Organization. (2012). Available online at: http://www.ncbi.nlm.nih.gov/books/NBK131942/ (Accessed October 26, 2025). 23586122

[17] B-Lynch C Coker A Lawal AH Abu J Cowen MJ. The B-Lynch surgical technique for the control of massive postpartum haemorrhage: an alternative to hysterectomy? Five cases reported. Br J Obstet Gynaecol. (1997) 104:372–5. doi: 10.1111/j.1471-0528.1997.tb11471.x, PMID: 9091019

[18] Committee on Practice Bulletins-Obstetrics. Practice bulletin no. 183: postpartum hemorrhage. Obstet Gynecol. (2017) 130:e168–86. doi: 10.1097/AOG.0000000000002351 28937571

[19] Kaoiean S. Successful Use of the B-Lynch Uterine Compression Suture in Treating Intractable Postpartum Hemorrhage after Cesarean Delivery in Rajavithi Hospital. J Med Assoc Thai. (2013) 96:1408–15. 24428089

[20] Sylvester HC Stringer M. Placental abruption leading to hysterectomy. BMJ Case Rep. (2017) 2017:bcr2016218349. doi: 10.1136/bcr-2016-218349, PMID: 29233830 PMC5728244

[21] Sunanda N Sruthi T Sheela SR. Feto-maternal outcome in patients with couvelaire uterus: a 3 year study in a tertiary care hospital in rural Karnataka, India. Int J Reprod Contracept Obstet Gynecol. (2018) 7:503–7. doi: 10.18203/2320-1770.ijrcog20170162

[22] Ditisheim A Sibai BM. Diagnosis and Management of HELLP Syndrome Complicated by Liver Hematoma. Clin Obstet Gynecol. (2017) 60:190–7. doi: 10.1097/GRF.0000000000000253, PMID: 28005587

[23] Rimaitis K Grauslyte L Zavackiene A Baliuliene V Nadisauskiene R Macas A. Diagnosis of HELLP Syndrome: A 10-Year Survey in a Perinatology Centre. Int J Environ Res Public Health. (2019) 16:109. doi: 10.3390/ijerph16010109, PMID: 30609811 PMC6339138

[24] Ch’ng CL Morgan M Hainsworth I Kingham JGC. Prospective study of liver dysfunction in pregnancy in Southwest Wales. Gut. (2002) 51:876–80. doi: 10.1136/gut.51.6.876 12427793 PMC1773454

[25] George JN Nester CM. Syndromes of Thrombotic Microangiopathy. N Engl J Med. (2014) 371:654–66. doi: 10.1056/NEJMra1312353, PMID: 25119611

[26] Haram K Svendsen E Abildgaard U. The HELLP syndrome: clinical issues and management. A review. BMC Pregnancy Childbirth. (2009) 9:8. doi: 10.1186/1471-2393-9-8, PMID: 19245695 PMC2654858

[27] Ngene NC Moodley J. Preventing maternal morbidity and mortality from preeclampsia and eclampsia particularly in low- and middle-income countries. Best Pract Res Clin Obstet Gynaecol. (2024) 94:102473. doi: 10.1016/j.bpobgyn.2024.102473, PMID: 38513504

[28] Chuliber F Gutiérrez A Kordys E Ross I Romañano V Lobo E . Disponibilidad y distribución de los recursos de hemostasia y trombosis en Argentina en el año 2022. Hema. (2024) 28:5–23. doi: 10.48057/hematologa.v28i1.577

[29] Riley DS Barber MS Kienle GS Aronson JK von Schoen-Angerer T Tugwell P . CARE guidelines for case reports: explanation and elaboration document. J Clin Epidemiol. (2017) 89:218–35. doi: 10.1016/j.jclinepi.2017.04.026 28529185

